# Impact of molecular classification on prognosis in children and adolescents with spinal ependymoma: Results from the HIT-MED database

**DOI:** 10.1093/noajnl/vdae179

**Published:** 2024-10-23

**Authors:** Lara Engertsberger, Martin Benesch, Martin Mynarek, Svenja Tonn, Denise Obrecht-Sturm, Thomas Perwein, Martina Stickan-Verfürth, Angela Funk, Beate Timmermann, Michael Bockmayr, Alicia Eckhardt, Alexander Claviez, Rolf-Dieter Kortmann, Markus J Riemenschneider, Torsten Pietsch, Brigitte Bison, Monika Warmuth-Metz, Kristian W Pajtler, Stefan Rutkowski, Ulrich Schüller

**Affiliations:** Division of Pediatric Hemato-Oncology, Department of Pediatrics and Adolescent Medicine, Medical University of Graz, Graz, Austria; Division of Pediatric Hemato-Oncology, Department of Pediatrics and Adolescent Medicine, Medical University of Graz, Graz, Austria; Mildred Scheel Cancer Career Center HaTriCS4, University Medical Center Hamburg-Eppendorf, Hamburg, Germany; Department of Pediatric Hematology and Oncology, University Medical Center Hamburg-Eppendorf, Hamburg, Germany; Department of Pediatric Hematology and Oncology, University Medical Center Hamburg-Eppendorf, Hamburg, Germany; Department of Pediatric Hematology and Oncology, University Medical Center Hamburg-Eppendorf, Hamburg, Germany; Division of Pediatric Hemato-Oncology, Department of Pediatrics and Adolescent Medicine, Medical University of Graz, Graz, Austria; Department of Particle Therapy, University Hospital Essen, West German Proton Therapy Centre Essen (WPE), West German Cancer Center (WTZ), Essen, Germany; Department of Particle Therapy, University Hospital Essen, West German Proton Therapy Centre Essen (WPE), West German Cancer Center (WTZ), Essen, Germany; Department of Particle Therapy, University Hospital Essen, West German Proton Therapy Centre Essen (WPE), West German Cancer Center (WTZ), German Cancer Consortium (DKTK), Essen, Germany; bAIome - Center for Biomedical AI, University Medical Center Hamburg-Eppendorf, Hamburg, Germany; Research Institute Children’s Cancer Center Hamburg, University Medical Center Hamburg-Eppendorf, Hamburg, Germany; Department of Pediatric Hematology and Oncology, University Medical Center Hamburg-Eppendorf, Hamburg, Germany; Research Institute Children’s Cancer Center Hamburg, University Medical Center Hamburg-Eppendorf, Hamburg, Germany; Department of Pediatric Hematology and Oncology, University Medical Center Hamburg-Eppendorf, Hamburg, Germany; Department of Pediatric and Adolescent Medicine, Pediatric Hematology and Oncology, University Hospital Magdeburg, Magdeburg, Germany; Department of Radiation Oncology, University of Leipzig, Leipzig, Germany; Department of Neuropathology, Regensburg University Hospital, Regensburg, Germany; Institute of Neuropathology, DGNN Brain Tumor Reference Center, University of Bonn Medical Center, Bonn, Germany; Neuroradiological Reference Center for the pediatric brain tumor (HIT) studies of the German Society of Pediatric Oncology and Hematology, University Hospital Würzburg (until 2020), University Augsburg, Faculty of Medicine (since 2021), Germany; Institute of Diagnostic and Interventional Neuroradiology, University Hospital Würzburg, Würzburg, Germany; Institute of Diagnostic and Interventional Neuroradiology, University Hospital Würzburg, Würzburg, Germany; Division of Pediatric Neurooncology, German Cancer Consortium (DKTK), German Cancer Research Center (DKFZ), Heidelberg, Germany; Department of Pediatric Oncology, Hematology and Immunology, Heidelberg University Hospital, Heidelberg, Germany; Hopp Children’s Cancer Center Heidelberg (KiTZ) and National Center for Tumor Diseases (NCT), Heidelberg, Germany; Department of Pediatric Hematology and Oncology, University Medical Center Hamburg-Eppendorf, Hamburg, Germany; Institute of Neuropathology, University Medical Center Hamburg-Eppendorf, Hamburg, Germany; Research Institute Children’s Cancer Center Hamburg, University Medical Center Hamburg-Eppendorf, Hamburg, Germany; Department of Pediatric Hematology and Oncology, University Medical Center Hamburg-Eppendorf, Hamburg, Germany

**Keywords:** DNA methylation, molecular type, *MYCN*-amplification, pediatric spinal ependymoma, radiotherapy

## Abstract

**Background:**

Ependymomas of the spinal cord are rare among children and adolescents, and the individual risk of disease progression is difficult to predict. This study aims to evaluate the prognostic impact of molecular typing on pediatric spinal cord ependymomas.

**Methods:**

Eighty-three patients with spinal ependymomas ≤22 years registered in the HIT-MED database (German brain tumor registry for children, adolescents, and adults with medulloblastoma, ependymoma, pineoblastoma, and CNS-primitive neuroectodermal tumors) between 1992 and 2022 were included. Forty-seven tumors were analyzed by DNA methylation array profiling. In 6 cases, HOXB13 and MYCN proteins were detected as surrogate markers for specific methylation classes. Ten patients had *NF2*-related schwannomatosis.

**Results:**

With a median follow-up time of 4.9 years, 5- and 10-year overall survival (OS) were 100% and 86%, while 5- and 10-year progression-free survival (PFS) were 65% and 54%. Myxopapillary ependymoma (SP-MPE, *n* = 32, 63%) was the most common molecular type followed by spinal ependymoma (SP-EPN, *n* = 17, 33%) and *MYCN*-amplified ependymoma (*n* = 2, 4%). One case could not be molecularly classified, and one was reclassified as anaplastic pilocytic astrocytoma. 5-year PFS did not significantly differ between SP-MPE and SP-EPN (65% vs. 78%, *P* = .64). *MYCN*-amplification was associated with early relapses (<2.3 years) in both cases and death in one patient. Patients with SP-MPE subtype B (*n* = 9) showed a non-significant trend for better 5 years-PFS compared to subtype A (*n* = 18; 86% vs. 56%, *P* = .15). The extent of resection and WHO tumor grades significantly influenced PFS in a uni- and multivariate analysis.

**Conclusions:**

Molecular typing of pediatric spinal ependymomas aids in identifying very high-risk *MYCN*-amplified ependymomas. Further insights into the molecular heterogeneity of spinal ependymomas are needed for future clinical decision-making.

## Lay Summary

Ependymomas are tumors that can develop in the spinal cord. While some can be cured with surgery, others may come back after being removed. The authors of this study wanted to see if there were specific genetic changes within these tumors that contributed to their growth back after surgical removal. To do this, they studied the genetic makeup of 83 spinal ependymomas in children and adolescents. Their results showed that certain genetic changes, for example in a gene called MYCN, were linked to a higher chance of tumors returning after treatment.

**Key Points**

- SP-MPE and SP-EPN are the most common molecular types of pediatric spinal ependymoma (ped-spEP).
- *MYCN*-amplification in ped-spEP is rare but may predict poor survival.
- Gross-total resection of ped-spEP independently increases progression-free survival.

**Importance of the Study**

The present study provides a large set of both clinical (*n* = 83) and methylation data (*n* = 47) on pediatric spinal ependymomas. Gross-total resection was confirmed to be the major clinical prognostic parameter. DNA methylation analysis is crucial to identify very high-risk *MYCN*-amplified ependymomas. Since the 2 main molecular types (SP-MPE and SP-EPN) do not allow risk stratification in our cohort, identifying molecular subtypes that correlate with the clinical course is of great importance. In this regard, we included the recently proposed subtypes of SP-MPE and identified a trend towards better survival of subtype B than A in this pediatric cohort. Further studies with higher patient counts are urgently needed to evaluate the prognostic impact of different adjuvant treatment strategies.

Ependymomas are neuroepithelial malignancies of the central nervous system (CNS) accounting for approximately 5% of pediatric CNS tumors.^1^ Primary ependymomas located in the spinal cord are very rare among children and adolescents, comprising about 13% of all pediatric ependymomas.^2^

Based on DNA methylation profiling, spinal ependymomas are currently classified into 4 distinct histologically and molecularly defined types: spinal myxopapillary ependymoma (SP-MPE), spinal ependymoma (SP-EPN), spinal subependymoma (SP-SE), and *MYCN*-amplified SP-EPN (SP-EPN-MYCN).^3^ In the latter, *MYCN*-amplification invariably drives an aggressive clinical course.^4,5^ Within the spinal compartment, these ependymoma types are characterized by characteristic histopathological features, but the differentiation of spinal ependymomas, myxopapillary ependymomas, and subependymomas by histology alone is sometimes difficult.^6^

Recently, the molecular type of SP-MPE was further divided into MPE-A and MPE-B.^7^ While MPE-A tumors occurred in younger patients (median age 27 years) and relapsed in 85% within 10 years, patients with MPE-B were older (median age 45 years) and had a significantly better outcome with a relapse rate of only 33% in 10 years. Papillary and tanycytic tumors typically belonged to MPE-A and MPE-B, respectively, whereas predominantly myxoid tumors appeared in both subtypes. MPE-A were enriched with tumors demonstrating *MGMT* promoter hypermethylation and increased copy number alterations, compared to MPE-B.^7^

According to the 2021 WHO Classification of Tumors of the CNS, spinal ependymomas can be categorized into CNS WHO grade 2 or 3 tumors.^3^ While CNS WHO grade 2 ependymomas are frequent and slowly growing neoplasms, CNS WHO grade 3 tumors are very rare, but aggressive and highly malignant.^8^ In contrast to previous WHO classifications, myxopapillary ependymoma is now considered CNS WHO grade 2 rather than 1 due to the relatively high risk of relapse. Still, myxopapillary ependymoma and subependymoma remain histopathologically defined tumor types, since, until the most recent WHO classification, there was no evidence that classification by molecular means would provide more clinical utility than the morphologic classification in these 2 tumor types.^3,9,10^

In general, the clinical outcome of pediatric spinal cord ependymomas is superior to that of intracranial ependymomas with a 5-year overall survival (OS) of 90%–100%^8,11,12^ versus 50%–64%,^13^ respectively. Progression-free survival (PFS) of pediatric spinal cord ependymomas declines progressively with time and varies between 70% and 90% at 5 years and 70% at 10 years.^8,11,12^ Poor outcome has been described with CNS WHO grade 3 ependymoma, although long-term survival is possible in this rare type.^8^ Especially pediatric myxopapillary ependymomas show a high tendency towards dissemination and a recurrence rate of up to 40% irrespective of the extent of resection.^14,15^ Nonetheless, reported OS rates are favorable extending from 85% to 100%.^8^

Treatment of patients with spinal ependymomas remains challenging due to the frequent infeasibility of gross total resection (GTR), the paucity of established treatment protocols, and the uncertainty regarding the therapeutic value of radiotherapy.^5^ Thus, therapeutic decisions must be made based on the individual risks of disease progression. However, these remain difficult to predict. Presently, the extent of resection is the strongest clinical predictor for PFS and OS. Neither age, sex, and tumor site nor histological grading had a significant influence on survival in a multi-institutional series including 29 children with spinal ependymomas.^11^ Data on the prognostic impact of molecular subtyping of spinal ependymomas in pediatric cohorts are currently missing.^6^

Furthermore, it is unclear if the spectrum of DNA methylation profiles in pediatric spinal ependymomas resembles that of adults. To date, one study aimed at the molecular characterization of pediatric spinal ependymomas and identified 18 pediatric ependymomas as SP-MPE and 5 as SP-EPN, but failed to classify further 4 cases. This finding points towards substantial differences between the molecular types of pediatric vs. adult spinal ependymoma.^16^

The present study aims to summarize the currently existing clinical information, including follow-up and treatment-related data, of pediatric patients with spinal cord ependymomas registered in the HIT-MED database (brain tumor registry for children, adolescents, and adults with medulloblastoma, ependymoma, pineoblastoma, and CNS-primitive neuroectodermal tumors) in Hamburg, Germany. In particular, we wanted to explore the role of molecular characterization for individual risk assessment and future treatment strategies.

## Materials and Methods

Eighty-two patients younger than 22 years with primary spinal ependymomas registered in the HIT-MED database between 1992 and 2022 as well as one additional patient, who was treated in the West German Proton Therapy Center in Essen (WPE), were included in this study. Twenty-eight tumors with available DNA methylation data had previously been included in Bockmayr et al.^7^ and Neyazi et al.^17^ All patients gave written informed consent according to the research proposals approved by the institutional review boards of the participating institutions (Clinical Ethics Committee at the University Medical Center Hamburg-Eppendorf, Ethics Committee at the Medical Faculty of the University Duisburg-Essen, Medical Ethics Committee at the Julius Maximilian University of Würzburg).

Baseline epidemiological characteristics (sex, age at diagnosis), tumor- and treatment-related data (primary tumor site, initial metastasis, neuropathological findings, cerebrospinal fluid (CSF) cytology, extent of resection, and radio-/chemotherapy), and outcome parameters were analyzed retrospectively. OS was defined as the time from initial diagnosis to death from any causes and PFS as the time from diagnosis to first relapse or progression. All data were extracted without direct personal identification. Treatment centers were contacted by the HIT-MED data management center to obtain the most recent follow-up information.

### Radiologic Staging

Preoperative craniospinal and postoperative spinal MRI was performed by local radiologists and centrally reviewed in 59 patients (71%) at the Neuroradiological Reference Center for the HIT Studies of the German Society of Pediatric Oncology and Hematology at the University Hospital Würzburg and, since 2021, at the University Hospital Augsburg, Germany.

### Surgical and Adjuvant Treatment

Surgery was the primary diagnostic and therapeutic approach in all patients. Surgical outcome was defined as GTR if no residual tumor mass was detectable in the postoperative MRI or—in case of missing postoperative MRI (*n* = 2)—if total tumor resection was stated in the surgical report. In the case of dissemination at diagnosis, only full resection of all visible tumor masses including metastasectomy was defined as GTR. Cases in which GTR was not achieved were defined as “less than GTR” (<GTR, including subtotal resection and partial resection) or biopsy. After surgery, the treating physician decided on an individual basis whether to add adjuvant radio- and/or chemotherapy.

### Histology

Tumor tissue specimens for histopathological examination were obtained by tumor resection or biopsy. Local neuropathologists determined the histopathological diagnosis according to the WHO Classification of Tumors of the CNS. Results from earlier editions were adopted to the 2021 WHO Classification by renaming 2016 WHO grade I myxopapillary ependymoma as “WHO grade 2, myxopapillary” and the 2016 WHO grade II spinal ependymoma “WHO grade 2, non-myxopapillary.” The central neuropathological review was carried out in 74 cases (89%) by the HIT Reference Centers for Neuropathology at the University of Bonn Medical Center and the University Medical Center Hamburg-Eppendorf, Germany.

CSF cytology was performed in 41 patients (49%).

### DNA Methylation Profiling

DNA methylation profiling had either been performed prior to this study or tumor material for DNA methylation analysis was requested by the HIT-MED data management center.

DNA was isolated from FFPE tissue using the ReliaPrep FFPE gDNA Miniprep System (Promega) according to the manufacturer’s instructions. About 100–500 ng DNA was used for bisulfite conversion by the EZ DNA Methylation Kit (Zymo Research). The DNA Clean & Concentrator-5 (Zymo Research) and the Infinium HD FFPE DNA Restore Kit (Illumina) were employed to clean and restore the converted DNA. Finally, Infinium BeadChip arrays (Illumina) were used to quantify the methylation status on an iScan (Illumina).

Raw IDAT files were uploaded to MolecularNeuropathology.org for classification. In total, 47 DNA methylation profiles were classified according to the Heidelberg Brain Tumor Classifier V12.5 (*n* = 44) or V11b4, if IDAT files of preexisting methylation analyses were not accessible (*n* = 3). This deviation in classifier scores was accepted since the methylation class predictions rarely differ between classifier versions V11b4 and V12.5 regarding spinal ependymomas.

### Immunohistochemistry

In cases of insufficient amounts of obtained tumor material for DNA methylation profiling, immunohistochemistry with antibodies against HOXB13 (Santa Cruz Biotechnology, sc-28333, 1:100 dilution, CC1 OptiView pretreatment) and MYCN (Cell Signaling, #51705, 1:100 dilution, CC1 standard UltraView pretreatment) proteins were used as surrogate markers for the methylation type. HOXB13 and MYCN were previously reported as robust biomarkers for SP-MPE^7^ and SP-EPN-MYCN,^5^ respectively. Immunohistochemistry was performed using paraffin sections in an automated Ventana staining system.

### SP-MPE Subtyping

SP-MPE were further classified into subtypes SP-MPE-A and SP-MPE-B by t-distributed stochastic neighbor embedding (t-SNE) analysis, using the t-SNE embeddings of MPE-A and MPE-B samples identified by Bockmayr et al.^7^ While seventeen SP-MPE had previously been included in Bockmayr et al.,^7^ we analyzed de novo eleven SP-MPE subtypes.

### Statistics

All statistical analyses were performed using SPSS version 28.0 (SPSS Inc.) and R^18^ version 4.1.3 (packages: “readxl,” “tidyverse,” “dplyr,” “tibble,” “gt,” “gtExtra,” “ggplot2,” “ggpubr,” “ggpattern,” “networkD3,” “survival,” “survminer,” “forestmodel,” “patchwork”). Fisher’s Exact Test was used to identify statistically significant differences in categorical frequencies. Univariate survival analysis was calculated using the Kaplan–Meier estimator and log-rank test for categorical variables or Cox regression for continuous variables. Multivariate survival analysis was performed with the Cox proportional hazards model. Schoenfeld residuals were used to check the proportional hazards assumption, Martingale residuals to assess nonlinearity, and Deviance residuals to examine influential observations. Variables not meeting the before-mentioned assumptions were removed from the multivariate model and variables with zero or few events were either grouped, if applicable, or removed. The goodness of fit was evaluated by the Likelihood ratio, Wald, and Score test as well as Harrell’s C statistic for concordance. *P* values <.05 were considered statistically significant.

## Results

### Cohort

In total, 89 patients with histologically confirmed spinal ependymomas were screened, five of whom were excluded due to age above 22 years (*n* = 4) or re-diagnosis as diffuse midline glioma on repeated histopathological review (*n* = 1). Additionally, DNA methylation profiling led to the reclassification of one tumor as anaplastic pilocytic astrocytoma (ANA-PA), which was therefore excluded as well.

**Figure 1.**
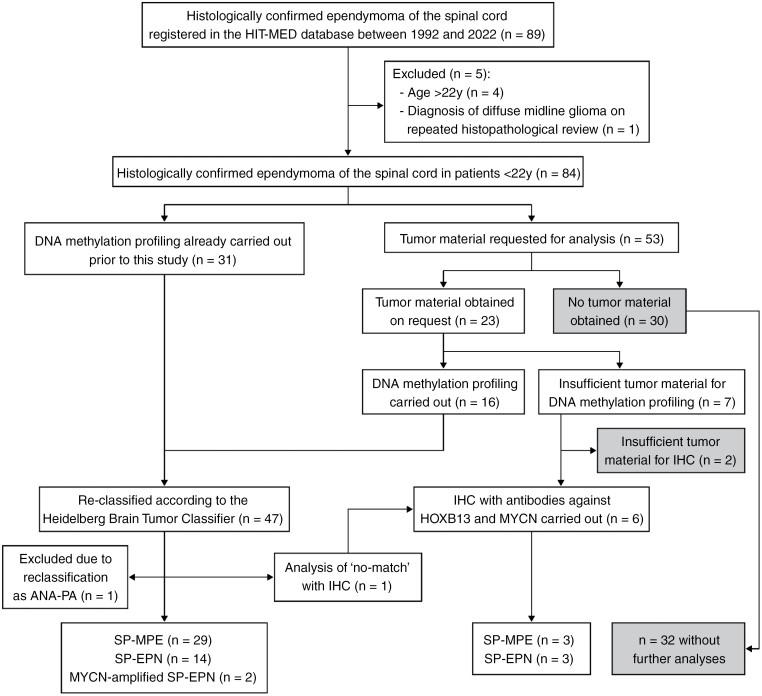
Consort diagram on the patient selection process and collection of DNA methylation profiles. In cases of insufficient tumor material for DNA methylation profiling, HOXB13 and MYCN protein expressions were used as surrogate markers for SP-MPE and to exclude SP-EPN-MYCN, respectively. Abbreviations: y, years; IHC, immunohistochemistry; ANA-PA, anaplastic pilocytic astrocytoma; SP-MPE, spinal myxopapillary ependymoma; SP-EPN, spinal ependymoma.

DNA methylation profiling had already been performed prior to this study in 31 cases. In the remaining cases, tumor material for DNA methylation analysis was requested by the HIT-MED data management center. Tumor samples were obtained from 23 patients and sufficient for DNA methylation profiling in 16 cases. In five cases with insufficient tumor material and in another case with no match in DNA methylation profiling, immunohistochemistry with antibodies against HOXB13 and MYCN proteins were used as surrogate markers for the methylation types of SP-MPE or SP-EPN-MYCN, respectively. In total, 52 spinal ependymomas were assigned to a methylation type. Ependymomas belonging to the DNA methylation type of SP-MPE were further classified into subtypes SP-MPE-A and -B. In 2 cases, subtyping was not possible as the corresponding IDAT files were unavailable (Figure 1).

### Clinical Characteristics

#### Total cohort.—

**Figure 2.**
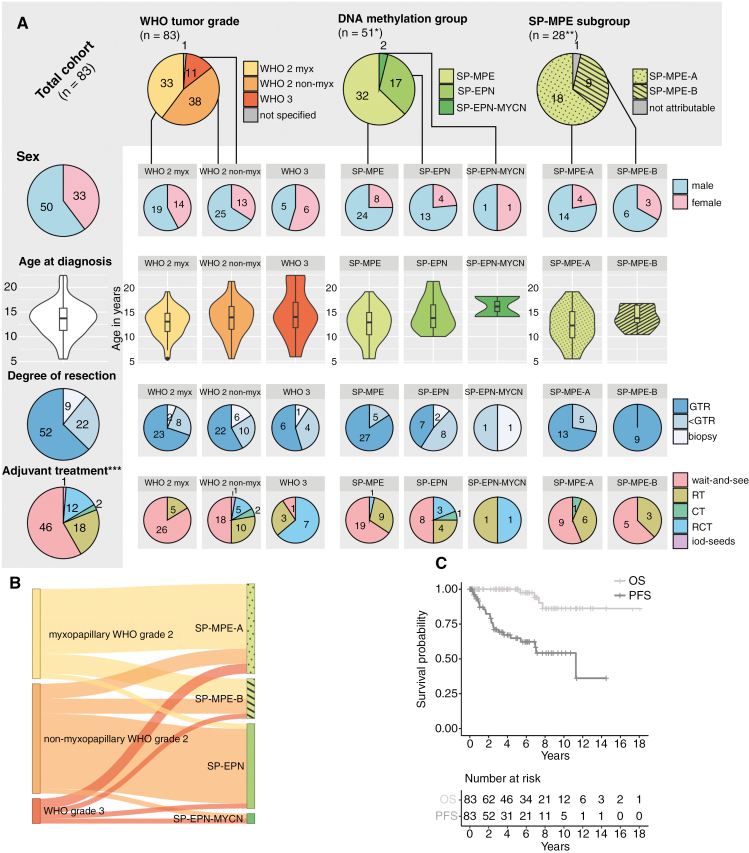
Cohort overview. **(A)** Patient characteristics. *One patient was excluded due to molecular reclassification of tumor material as anaplastic pilocytic astrocytoma (ANA-PA). **SP-MPE subtyping was impossible in those classified as SP-MPE by immunohistochemistry (*n* = 2) and was missing in *n* = 2 because of unavailable IDAT files. ***In 4 patients, initial adjuvant treatment was not specified. **(B)** Allocation of histological WHO tumor grades to DNA methylation profiles. **(C)** Overall and progression-free survival in pediatric spinal ependymoma. Abbreviations: *n*, number; myx, myxopapillary; SP-MPE, spinal myxopapillary ependymoma; SP-EPN, spinal ependymoma; SP-EPN-MYCN, *MYCN*-amplified SP-EPN; SP-MPE-A, SP-MPE subtype A; SP-MPE-B, SP-MPE subtype B; GTR, gross total resection; <GTR, less than GTR (subtotal or partial resection); RT, radiotherapy; CT, chemotherapy; RCT, radiochemotherapy; OS, overall survival; PFS, progression-free survival.

Baseline clinical characteristics are summarized in Figure 2. Median age at diagnosis was 13.7 years (range 5.5–22.4). Of the 83 patients included in the final analysis, 50 (60%) were male and 33 (40%) female. Ten children (12%) were diagnosed with *NF2*-related schwannomatosis (formerly neurofibromatosis type 2 [NF2]) due to clinical characteristics and/or a germline mutation in the *NF2* gene. Disseminated disease at the time of diagnosis (M+) was noted in 14 patients (17%), 2 of whom (2%) had intracranial metastases. The most dominant primary tumor site was the lumbar (*n* = 23, 28%) followed by the thoracolumbar (*n* = 13, 16%), cervical (*n* = 11, 14%), and cervicothoracic region (*n* = 10, 12%). A detailed list of tumor sites is given in Supplementary Table S1. Thirty-three patients (40%) were diagnosed with CNS WHO grade 2 myxopapillary ependymoma, 38 patients (46%) with CNS WHO grade 2 non-myxopapillary ependymoma, and 11 patients (13%) with CNS WHO grade 3 ependymoma (histopathological grading not specified in *n* = 1).

#### DNA methylation types.—

SP-MPE was the most common molecular type (*n* = 32, 63%) followed by SP-EPN (*n* = 17, 33%). Two cases (4%) were *MYCN*-amplified SP-EPN. One sample (1%) had no match for any DNA methylation type in classifier V12.5 but was HOXB13 positive, showed distinct features of a myxopapillary ependymoma with an SP-MPE-A subtype in the t-SNE visualization, and was therefore assigned as SP-MPE (Figure 1).

In this pediatric cohort, SP-MPE subtype A was more common with 64% (*n* = 18) compared to subtype B with 32% (*n* = 9). One SP-MPE case was neither attributable to subtype A nor B. Myxopapillary grade 2 ependymomas as by histology, molecularly classified predominantly as SP-MPE (*n* = 20/21, SP-EPN: *n* = 1), whereas non-myxopapillary grade 2 ependymomas clustered into SP-MPE (*n* = 8/24) and SP-EPN (*n* = 15/24). One non-myxopapillary grade 2 ependymoma had an *MYCN* amplification. Grade 3 ependymomas molecularly classified either as SP-MPE (*n* = 3/5), SP-EPN (*n* = 1/5), or SP-EPN-MYCN (*n* = 1/5; Figure 2B).

Initial dissemination was observed in 8/32 (25%) SP-MPE, 1/2 (50%) SP-EPN-MYCN, and none of SP-EPN. While one-third of the patients with SP-MPE subtype A were initially metastasized (*n* = 6/18), M + occurred in only 13% (*n* = 1/8) of subtype B (*P* = .28).

### Initial Staging

Preoperative MRI of the spinal cord was performed in all patients. However, only 60 patients (74%) received complete initial craniospinal MRI (incomplete: *n* = 21; unknown: *n* = 2). Postoperative spinal MRI was carried out in 81 patients (98%). Fifty-nine patients (71%) additionally underwent central radiologic review. Staging according to standardized MRI criteria was impossible in 23 cases (39%), mostly due to an excess of time between the surgery and postoperative MRI, which complicates the differentiation of scar tissue from a possible residual tumor mass. CSF cytology was documented in 41 patients (49%, M+: *n* = 5/41), and the results were negative in all cases.

### Surgical Procedures

The primary surgical intervention included tumor resection in 69 (83%) and diagnostic biopsy in 14 patients (17%). Seventy patients underwent single surgery, and 13 patients underwent a second surgery due to prior incomplete surgery (5 after biopsy, 8 after <GTR). Second surgery after <GTR resulted in GTR in 5/8 cases and in GTR of the primary tumor with remaining metastases in 2/8. The median time to second surgery was 56 days (range 2–125 days). Another patient (not included above) did not receive second surgery (GTR) until 210 days after diagnosis, when he had already completed radiochemotherapy and was therefore included in the <GTR group for analysis.

Of the 9 patients who underwent only biopsy without further surgery, one was diagnosed with NF2, and another received second surgery after completion of radiochemotherapy (267 days after biopsy). In another 2 cases, the tumor was deemed unresectable, whereas in the remaining patients, the reason for omitting tumor resection was unknown.

Taken together, GTR was achieved in a total of 52 patients and <GTR (subtotal or partial resection) in 22 patients before the initiation of adjuvant treatment. Subsequent surgery due to relapse or disease progression was necessary in 13 patients.

### Adjuvant Radiotherapy and/or Chemotherapy

Adjuvant treatment was started after a median of 41 days (range 0–222 days) following primary surgical procedures. In seven patients, the time span from resection to adjuvant treatment as first-line therapy exceeded 90 days (NF2: *n* = 2, prolonged interval to second surgery before adjuvant treatment: *n* = 2, reason unknown: *n* = 3). Radiotherapy alone was used in 18 patients (proton radiation: *n* = 4), chemotherapy alone in 2, combined radiochemotherapy in 12 (proton radiation: *n* = 1), and iodine seed implantation (65 Gy) in one. The latter patient was excluded from the radiotherapy group. Forty-six patients received no adjuvant treatment (wait-and-see approach; adjuvant treatment unknown in *n* = 4).

Radiotherapy was administered either locally (*n* = 22; M+: *n* = 1) at a median cumulative dose of 50.4 Gy (range 48.6–68.0 Gy) or craniospinal (CSI, *n* = 6; M+: *n* = 5) at a median craniospinal axis dose of 35.2 Gy (24.0–40.0) and boost dose of 48.7 Gy (40.0–55.0, irradiated area not reported in *n* = 2). Additional spinal doses in CSI were given in 2 patients (38 and 44.8 Gy). 21 patients received conventional (M+: *n* = 4) irradiation with a daily single dose of 1.6–1.8 Gy, and 6 patients hyperfractionated irradiation (M+: *n* = 2, fractionation scheme not reported in *n* = 3) with 2 × 1.0 Gy per day. During radiotherapy, single-agent vincristine was administered to 7 patients and single-agent carboplatin to one patient.

Regarding the use of CSI, 5 of the 6 patients receiving CSI had WHO grade 3 tumors, one of which had an *MYCN* amplification, and 5 of the 6 CSI patients were M+. Five of the six CSI patients progressed, whereas only 7 of the 22 patients progressed after local radiotherapy (Supplementary Figure S1).

Several different chemotherapy regimens were used based on the HIT protocols that were active at the time of treatment (Supplementary Table S2). The most frequently administered chemotherapy regimen was modified SKK including one block of cyclophosphamide/vincristine followed by one block of carboplatin/etoposide per cycle (*n* = 8/14). Two patients received high-dose chemotherapy with autologous stem cell rescue, one during initial treatment and the other after multiple relapses. For relapsing tumors, various additional antineoplastic agents were administered, including tamoxifen/isotretinoin, bevacizumab, valproic acid, and melphalan.

### Adjuvant Therapy According to Extent of Resection, Tumor Grade, and Methylation Type

Adjuvant treatment was highly heterogeneous depending on the extent of resection, tumor grade, and molecular type (Figure 2A). Of all 52 patients, who underwent primary GTR, 16 (31%) received primary adjuvant treatment (data missing in *n* = 3). Contrarily, 9 (41%) of 22 patients with <GTR were treated with adjuvant therapy (data missing in *n* = 1). Eight (89%) of the 9 patients who were only biopsied were given adjuvant treatment (Chi-Square Test: *P* < .001).

### Clinical Course and Survival

#### Overall cohort.—

With a median follow-up (FU) time of 4.9 years (range 0.1–18.1), 79 patients (95%) were still alive at the last FU. For all pediatric spinal ependymomas, the 5- and 10-year OS was 100% and 86% [95% confidence interval: 73–99], respectively. Overall, 26 patients (31%) developed a relapse or progressive disease (PD; Table 1). At the time of diagnosis, 5-year PFS was 65% [54–77], whereas 10-year PFS was 54% [39–70] (Figure 2C). Regarding survival after the first PD, 5-year OS fell to 88.8% [73.8–100], while 2- and 5-year PFS were 59.8% [39.0–80.6] and 39.3% [17.7–60.9], respectively. Intracranial metastases were observed in 5 patients (SP-EPN-MYCN: *n* = 2, SP-MPE-A: *n* = 2, SP-EPN: *n* = 1). Fourteen patients had multiple relapses and 4 of them died after a mean of 6.8 years (range 5.4–7.7) after diagnosis.

**Table 1.** Relapses/Progressions After Initial Therapy Subdivided Into Tumor Classification and Treatment Strategies

		WHO tumor grading					
		WHO grade 2, myxopapillary		WHO grade 2, non-myxopapillary		WHO grade 3	
The extent of resection	Adjuvant therapy	PD (*n*/total)	%	PD (*n*/total)	%	PD (*n*/total)	%
GTR	WS	8/19	42.1%	1/13	7.7%		
	RT	0/2	0.0%	1/7	14.3%	0/3	0.0%
	CT			0/1	0.0%		
	RCT					1/3	33.3%
<GTR	WS	3/6	50.0%	1/5	20.0%	1/1	100.0%
	RT	1/2	50.0%	0/1	0.0%		
	RCT			2/3	66.7%	2/3	66.7%
Biopsy	WS	1/1	100.0%				
	RT	0/1	0.0%	1/2	50.0%		
	CT			0/1	0.0%		
	RTC			2/2	100.0%	1/1	100.0%
		Methylation group					
		SP-MPE		SP-EPN		SP-EPN-MYCN	
Extent of resection	Adjuvant therapy	PD (*n*/total)	%	PD (*n*/total)	%	PD (*n*/total)	%
GTR	WS	5/16	31.3%	0/3	0.0%		
	RT	1/7	14.3%	0/2	0.0%		
	CT			0/1	0.0%		
	RCT	0/1	0.0%	1/1	100.0%		
<GTR	WS	2/3	66.7%	1/5	20.0%		
	RT	1/2	50.0%	0/1	0.0%		
	RCT			1/1	100.0%	1/1	100.0%
Biopsy	RT			0/1	0.0%	1/1	100.0%
	RCT			1/1	100.0%		

WHO tumor grading is not specified in *n* = 1, not specified adjuvant treatment in *n* = 4, and seeds-implant is not shown (*n* = 1). Abbreviations: GTR, gross total resection; <GTR, less than GTR (subtotal or partial resection); WS, wait-and-see strategy; RT, radiotherapy; CT, chemotherapy; RCT, radiochemotherapy; SP-MPE, spinal myxopapillary ependymoma; SP-EPN, spinal ependymoma; PD, relapse or progressive disease.

**Figure 3.**
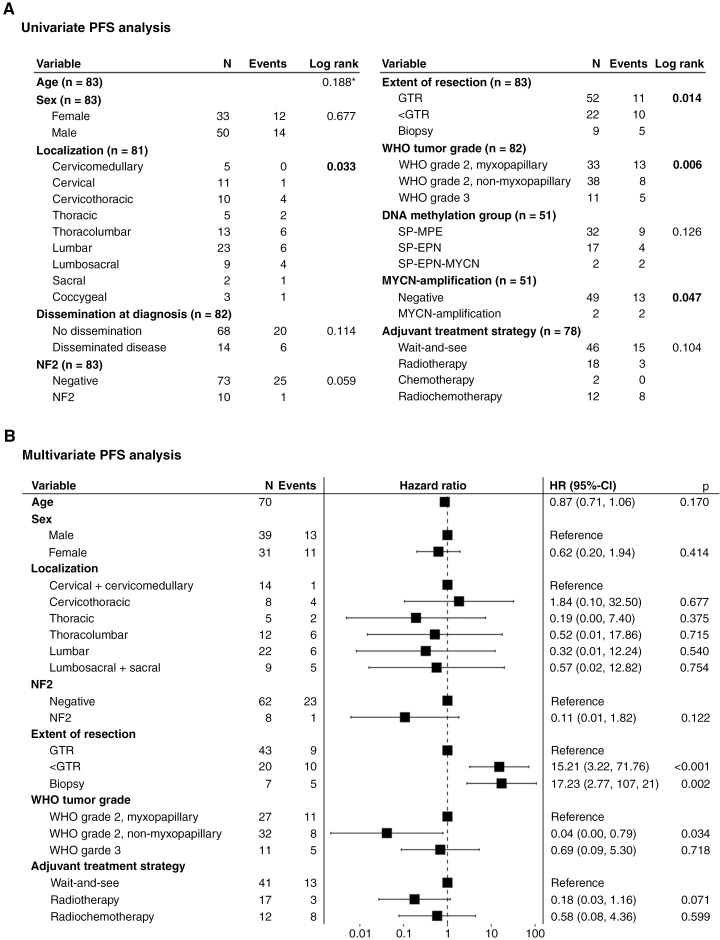
(A) Univariate and (B) multivariate progression-free survival analysis in pediatric spinal ependymoma. Univariate survival analysis was calculated using the Kaplan–Meier estimator if not stated otherwise. *calculated using Cox Regression. Multivariate survival analysis was performed with Cox Regression. DNA methylation was excluded from the final multivariate model as it did not meet the proportional hazards assumption and induced a strong selection bias due to the reduction of total patient count to *n* = 41. Dissemination at diagnosis was also removed because it did not meet the proportional hazards assumption. Subvariables with few or zero events were either combined if applicable (cervicomedullary and cervical, lumbosacral and sacral tumor localization) or excluded from the final model (coccygeal tumor localization, *MYCN*-amplification, chemotherapy). Cases excluded due to missing values: *n* = 13. Likelihood ratio test: χ^2^ = 45.21 (*P* = .00004), Wald test: χ^2^ = 28.44 (*P* = .01), Score test: χ^2^ = 43.35 (*P* = .00008), Concordance = 0.836. Abbreviations: PFS, progression-free survival; NF2, *NF2*-related schwannomatosis; GTR, gross total resection; <GTR, less than GTR (subtotal or partial resection); HR, hazard ratio.

**Figure 4.**
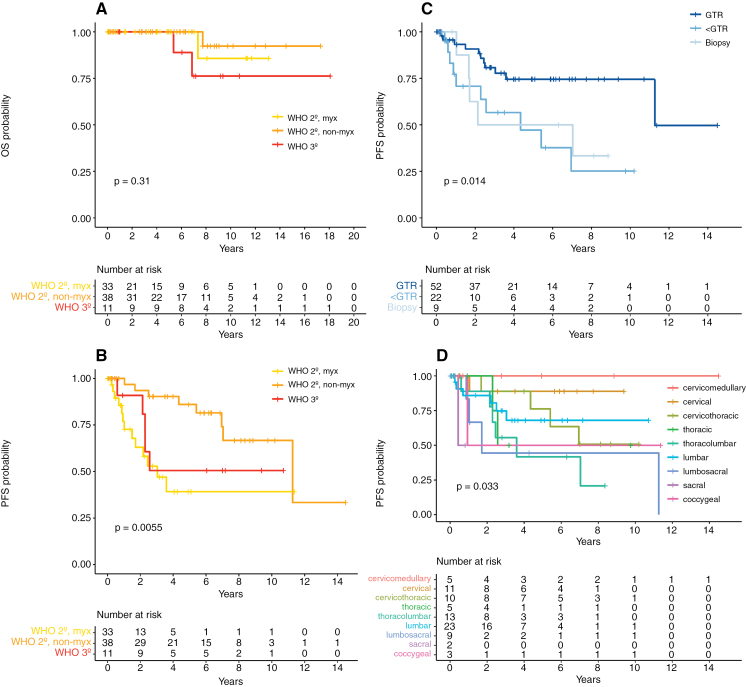
General prognostic factors in pediatric patients with spinal ependymoma. **(A)** OS stratified by WHO tumor grade. Factors significantly influencing PFS in the univariate analysis included **(B)** WHO tumor grade, **(C)** extent of resection, and **(D)** tumor localization. Involvement of caudal spinal segments resulted in worse PFS (*P* = .033). PFS was 100% in cervicomedullary tumor localization, 89% [95% CI: 67.9–100] in cervical, 76% [46.6–100] in cervicothoracic, 50% [0–100] in thoracic, 42% [7.1–76.3] in thoracolumbar, 68% [45.8–90.2] in lumbar, 44% [0–88.8] in lumbosacral, 50% [0–100] in sacral, and 50% [0–100] in coccygeal tumor localization. Abbreviations: OS, overall survival; PFS, progression-free survival; GTR, gross total resection; < GTR, less than GTR (subtotal or partial resection); myx, myxopapillary; y, year; CI, confidence interval.

None of the tested variables had an impact on OS in univariate analysis (Figure 3A, 4A). PFS was significantly influenced by WHO tumor grade (Figure 4B), the extent of resection (Figure 4C), and tumor localization (Figure 4D; Supplementary Table S3-5). However, in multivariate analysis, the extent of resection and WHO tumor grade remained the only 2 significant factors independently influencing PFS (Figure 3B). 5-year PFS 75% for GTR [60–89], 47% for <GTR [20–74], and 51% for biopsy [15–85] (*P* = .014); 39% for myxopapillary WHO grade 2 [16–62], 86% for non-myxopapillary WHO grade 2 [73–99], and 51% for WHO grade 3 ependymoma [19–82] (*P* = .006).

#### Impact of methylation type on prognosis in pediatric spinal ependymoma.—

**Figure 5.**
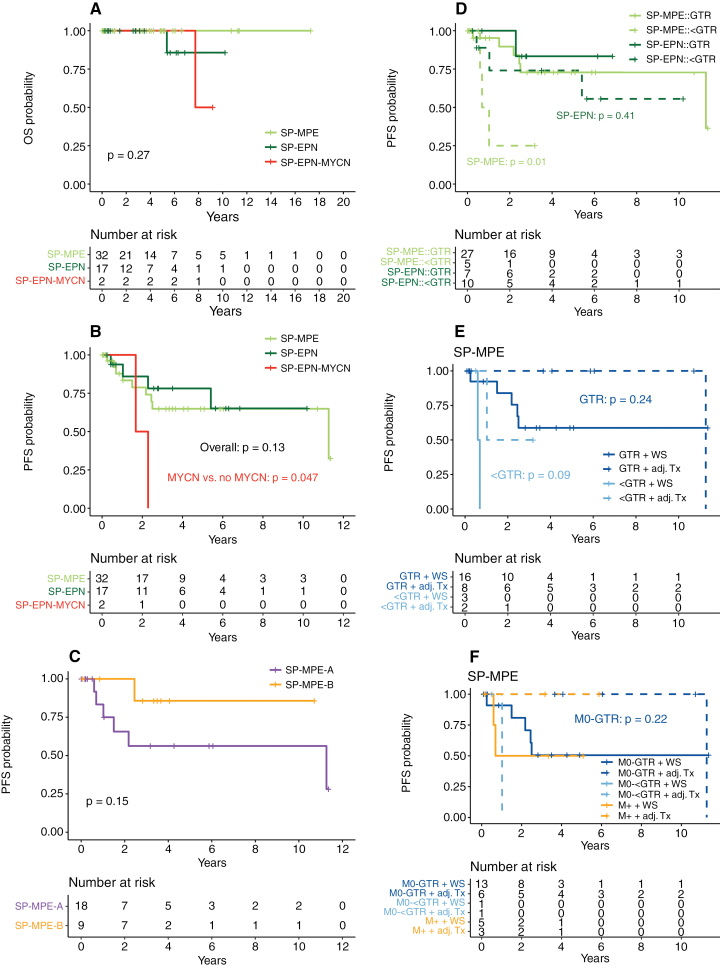
Impact of methylation groups on prognosis in pediatric spinal ependymoma. **(A)** OS and **(B)** PFS stratified by methylation group. PFS did not differ between methylation groups, whereas *MYCN* amplification significantly impacted PFS. **(C)** Influence of SP-MPE subtypes A and B on PFS. **(D)** PFS is significantly impacted by the extent of resection in SP-MPE but not in SP-EPN. **(E)** PFS analysis for the use of adjuvant treatment in SP-MPE after GTR or <GTR, and **(F)** after M0-GTR, M0-<GTR, or M+. Abbreviations: OS, overall survival; PFS, progression-free survival; SP-MPE, spinal myxopapillary ependymoma; SP-EPN, spinal ependymoma; SP-EPN-MYCN, *MYCN*-amplified spinal ependymoma; SP-MPE-A, SP-MPE subtype A; SP-MPE-B, SP-MPE subtype B; GTR, gross total resection; < GTR, less than GTR (subtotal or partial resection); WS, wait-and-see strategy; adj. Tx, adjuvant treatment; M0-GTR, GTR in the non-metastatic state; M0-<GTR, <GTR in the non-metastatic state; M+, metastatic state irrespective of the extent of resection.

Nine (28%) and 4 (31%) patients with molecularly defined SP-MPE or SP-EPN relapsed or progressed (*P* = n.s.). Neither OS nor PFS differed significantly between SP-MPE and SP-EPN (Figure 5A, 5B). Both patients with SP-EPN-MYCN progressed (5-year PFS [*MYCN*-amplification] 0% vs. [no *MYCN*-amplification] 70% [54–85], *P* = .047, Figure 5B). Dissemination at the time of diagnosis had no influence on survival regarding the subgroup of patients in which a methylation profile was available. Comparison of survival of patients with SP-MPE subtype A and B revealed a trend towards better PFS of subtype B than subtype A, similar to the findings in adults^7^ (5-year PFS 86% [59–100] vs. 56% [27–86], *P* = .152, Figure 5C).

In SP-MPE, the extent of resection significantly influenced PFS (5-year PFS [GTR] 73% [52–94] vs. [< GTR] 25% [0–68], *P* = .012), whereas this association was not significant in SP-EPN (Figure 5D). In patients with SP-MPE, there was a slight trend towards improved PFS with adjuvant therapy (radiotherapy or radiochemotherapy) compared to a wait-and-see approach. This trend remained consistent when differentiating between GTR and <GTR (Figure 5E) or between GTR in non-disseminated state (M0-GTR), <GTR in non-disseminated state (M0-<GTR), and disseminated state irrespective of the extent of resection (M+; Figure 5F). This pattern in favor of adjuvant therapy was not evident in patients with SP-EPN (Supplementary Figure S2). However, these findings must be interpreted with caution as they are based on only small numbers of patients in each subgroup.

#### NF2-associated ependymoma.—

NF2-associated ependymomas were predominantly located in the cervical spinal cord (cervicomedullary: *n* = 2, cervical: *n* = 5, cervicothoracic: *n* = 2, thoracolumbar: *n* = 1). At the time of diagnosis, no NF2-related tumor was disseminated. The most dominant WHO tumor grade was non-myxopapillary WHO grade 2 (*n* = 9), whereas one patient suffered from a WHO grade 3 ependymoma. All 6 NF2 patients with available DNA methylation profiles of their tumors were assigned to SP-EPN. GTR was achieved in 5 patients (50%), whereas 4 patients (40%) received <GTR, and 1 patient (10%) was biopsied only. A wait-and-see approach was the most common treatment strategy after resection (*n* = 6), followed by radiotherapy (*n* = 2) and chemotherapy (*n* = 1); in one patient the adjuvant treatment strategy was not specified. Progression occurred in only one patient after initial observation. Upon PD, a wait-and-see approach was again followed, and the tumor mass remained stable throughout the follow-up period. OS and PFS did not differ between patients with and without NF2.

## Discussion

This study presents a large compilation of clinical, histopathological, and DNA methylation data of children and adolescents with spinal ependymomas. SP-MPE is the most common molecular type of pediatric spinal ependymoma with 63% (*n* = 32/51) followed by SP-EPN with 33% (*n* = 17/51). Interestingly, the proportion of SP-MPE subtypes A to B of 64% to 32% (missing: *n* = 1) in this pediatric cohort was inverted compared to that of the adult population of 40% to 60%, reported by Bockmayr et al.^7^ DNA methylation analysis led to the identification of 2 SP-EPN-MYCN associated with a high risk for relapse and poor survival. Thus, our work supports previous reports on this aggressive form of spinal ependymoma driven by *MYCN* amplification.^4,5^ So far, spinal subependymoma (SP-SE) has not observed in children or adolescents, neither in our nor in other cohorts.^6,16^

SP-MPE and SP-EPN do not differ in terms of overall and progression-free survival probability. However, we observed a trend towards improved PFS in SP-MPE subtype B compared to subtype A in accordance with results in the adult population showing relapse rates of 85% vs. 33% after 10 years in subtype A vs. subtype B, respectively.^7^ In addition, subtype A might be more frequently disseminated at the time of diagnosis (*n* = 6/18) than subtype B (*n* = 1/8; *P* = .28).

In this pediatric cohort, one-third of the molecularly defined SP-MPE did not show typical myxopapillary histology, a notable contrast to the 17% reported in Pajtler et al.’s publication.^6^ However, the latter cohort consisted of 19 adults and only 1 child with SP-MPE (age not given: *n* = 6).^6^ In our study, SP-MPE subtypes A and B showed comparable histological inhomogeneity. This suggests significant diversity among the presently recognized subtypes of SP-MPE, particularly in pediatric patients, prompting the need for further research into the heterogeneity of the existing molecular types of pediatric spinal ependymoma.

Atypical extradural localization of myxopapillary ependymomas in the sacrococcygeal region was observed in 3 patients (Supplementary Table S1). DNA methylation analysis performed in 2 of the 3 patients consistently revealed an MPE subtype A. Myxopapillary ependymomas in this rare localization are initially often misinterpreted as other tumor entities, highlighting the diagnostic importance of DNA methylation analysis. A case study on one of these patients was previously published by Claviez et al.^19^

In terms of risk assessment, we identified the extent of resection, localization, and WHO tumor grading as significant predictors of PFS. In the multivariate analysis, both the extent of resection and WHO tumor grade remained significant.

GTR has already been proposed as a main prognostic parameter in pediatric spinal ependymomas. However, its superiority to subtotal or partial resection did previously not reach significance due to a smaller patient count.^11^ Here, we confirm the importance of GTR in the treatment of pediatric spinal ependymomas as the single clinical independent risk factor of PFS. Especially after diagnosis of spinal ependymoma via biopsy, GTR should be attempted in a second surgical intervention, if possible, without mutilation.

Regarding the risk assessment by histological grading, the non-myxopapillary WHO grade 2 conveyed a significantly lower risk of progression compared to the myxopapillary WHO grade 2 or WHO grade 3, which aligns with the relatively high risk of relapse for myxopapillary ependymomas.^9,10^

The diagnostic work-up of pediatric spinal ependymomas was highly heterogeneous and often incomplete. Only 74% of patients in our cohort received complete initial MRI, and metastatic status was unknown in 26% at the time of diagnosis. However, dissemination was ruled out in many cases retrospectively. For a comprehensive assessment of metastatic status, the initial MRI must include both cranial and spinal MRI, with the latter capturing the entire dural sac. Moreover, postoperative staging according to standardized MRI criteria was impossible in nearly 40% due to surgery-induced change and/or excess time between the surgery and postoperative MRI. Postoperative blood, hemostatic materials, edema, and unspecific postoperative contrast enhancement are more common after spinal surgery than after cranial CNS surgery. The early postoperative MRI should be conducted within 72 hours following surgery, best on the first postoperative day. Otherwise, granulation or scar tissue cannot be differentiated from a possible residual tumor mass. Comparability with the preoperative MRI is essential for the detection of residual tumors.^20^ Furthermore, reference neuroradiology and histology should be mandatory in all patients with spinal cord ependymomas.

The use of adjuvant radiotherapy in pediatric patients with spinal ependymomas is still a matter of debate. Avoiding radiotherapy is of particular importance in children, who otherwise may suffer from considerable radiation-induced late effects. Even though patients with myxopapillary ependymomas and their corresponding DNA methylation type of SP-MPE have a very good OS, they relapse very frequently even after M0-GTR. Thus, radiotherapy is considered especially in this type of spinal ependymoma.

A systematic review from 2013 points towards the benefit of adjuvant radiotherapy in the treatment of myxopapillary ependymomas in children, with recurrence rates of 65% after GTR and no radiotherapy compared to 16.7% after <GTR and radiotherapy.^15^ A 2019 published retrospective multicenter study in 28 children with spinal ependymomas, including 15 myxopapillary ependymomas, showed a benefit of radiotherapy delivered to patients with GTR, but no such advantage in patients who received <GTR; however, based on 6 patients only.^21^ Also, a recent study from 2021 on limited-volume proton radiation in twelve pediatric patients with disseminated myxopapillary spinal ependymomas reported a 5-year PFS of 92% with only out-of-field recurrence and no in-field recurrences.^22^ Regarding the molecular type of SP-MPE and its implications for treatment, data are scarce even in adult cohorts. One study from 2018 included 29 patients with SP-MPE, of whom only 2 were treated with radiotherapy, while 27 did not receive any adjuvant therapy. With only 6 progressions in 29 patients with SP-MPE (21%), progressions were rarer than in our pediatric cohort.^23^ Data on the use of radiotherapy in pediatric SP-MPE did not exist up to this date.

Our data suggest an advantage of radiotherapy in pediatric SP-MPE, regardless of the extent of resection or metastatic status. However, our findings lack statistical significance. The limited number of our data also precludes drawing conclusions regarding the efficacy of radiotherapy in the treatment of the first PD. Thus, the indication for radiotherapy in SP-MPE cases should be made individually considering factors such as age, feasibility of re-operation, and the family’s need for safety.

Concerning the extent of the radiation field in SP-MPE, PD outside the irradiated field occurred in 2/7 locally irradiated, initially non-metastatic SP-MPE patients (Supplementary Figure S1D). Bearing in mind the low number of cases, this observation implies the use of extended field or CSI in the treatment of SP-MPE, aligning with findings of other adult and pediatric series.^24,25^ Intracranial metastases were observed in only 2/32 SP-MPE patients (both subtype A), suggesting spinal irradiation as a brain-sparing alternative over CSI. However, both PDs occurred not only outside the irradiated field but also at the original tumor side, which indicates that craniospinal irradiation may not have been effective as well.

As patients with SP-MPE subtype B show an insignificant tendency towards better PFS than patients with subtype A, further studies with larger patient cohorts are needed to explore the possibility of avoiding radiotherapy in patients with SP-MPE-B. In general, presently, the indication for postoperative radiotherapy considering the new molecular classification cannot clearly be defined.

In patients with SP-EPN, a trend toward better PFS using a wait-and-see strategy was observed irrespective of the extent of resection. However, this trend is most likely a reflection of poorer initial conditions in those who received adjuvant treatment, including <GTR, biopsy, and diagnosis of histological WHO grade 3 ependymoma. Also, 6/17 SP-EPN was observed in NF2 patients, in whom it is known that radiotherapy should be avoided and that SP-EPN has more benign courses.

As *MYCN*-amplification conveys a high risk of early relapse and worse OS, patients with SP-EPN-MYCN should undergo adjuvant treatment, in particular, prophylactic craniospinal irradiation because of a high propensity for spinal seeding.^26^

Patients with the tumor predisposition syndrome NF2 present in 18-53% with spinal cord ependymomas.^27^ All 6 NF2 patients within our cohort, of whom DNA methylation profiles were available, were reclassified as SP-EPN. Since most spinal cord ependymomas in NF2 patients do not cause any symptoms even for prolonged periods of time, tumor resection should be reserved for patients developing neurological symptoms or tumor progression. In contrast, a watchful waiting strategy seems to be the most appropriate approach for asymptomatic patients.^27^ In our cohort, NF2 patients did not statistically differ in OS or PFS from those without NF2; however, Neyazi et al. recently reported that germline or sporadic *NF2* mutations are linked to a significantly reduced PFS among patients with SP-EPN.^17^

## Conclusions

This retrospective study expands our current knowledge of spinal ependymomas in childhood and adolescence due to the correlation of clinical and methylation data. DNA methylation profiling represents a useful tool in the neuropathological diagnosis of spinal ependymoma and can help to identify very high-risk SP-EPN-MYCN. However, differentiation between SP-EPN and SP-MPE does not segregate patients into distinct risk groups. Given the high risk of recurrence in SP-MPE patients with <GTR or M+, radiotherapy should be considered. Because intracranial metastases are seldom observed in this entity, focal (M0) or spinal (isolated spinal metastasis) radiotherapy should be considered. Given the better prognosis of M0-GTR SP-MPE, the indication for radiotherapy needs to be established individually. Still, larger cohorts and further investigations of methylation class heterogeneity and therapy options in pediatric spinal ependymomas are needed to complete the basis for future clinical decision-making.

## Supplementary Material

vdae179_suppl_Supplementary_Materials

## Data Availability

The methylation data are deposited at the GEO repository GSE276972.
