# Supplementary material for: Impact of molecular classification on prognosis in children and adolescents with spinal ependymoma: Results from the HIT-MED database
Source: Neurooncol Adv. 2024 Oct 23;6(1):vdae179. doi: 10.1093/noajnl/vdae179 (PMC11662162; doi:10.1093/noajnl/vdae179)
Supplement: vdae179_suppl_Supplementary_Materials [file vdae179_suppl_Supplementary_Materials.docx]

**Supplementary material**

Supplementary Table S1

Supplementary Figure S1

Supplementary Table S2

Supplementary Table S3

Supplementary Table S4

Supplementary Table S5

Supplementary Figure S2

Supplementary Figure S3

|  | **No. of Patients** | **% of total** |
| --- | --- | --- |
| No. of patients | 83 |  |
| No. of patients with NF2 | 11 | 13.3% |
| **Sex** | | |
| Male | 50 | 60.2% |
| Female | 33 | 39.8% |
|  | **Years** | **Range in years** |
| Median age at diagnosis | 13.7 | 5.5-22.4 |
| Median follow-up time | 4.9 | 0.1-18.1 |
|  | **No. of Patients** | **% of total** |
| **Primary tumor site** (missing = 2) | | |
| Craniocervical | 5 | 6.2% |
| Cervical | 11 | 13.6% |
| Cervicothoracic | 10 | 12.3% |
| Thoracic | 5 | 6.2% |
| Thoracolumbar | 13 | 16.0% |
| Lumbar | 23 | 28.4% |
| Lumbosacral | 9 | 11.1% |
| Sacral | 2 | 2.5% |
| Coccygeal | 3 | 3.7% |
| **Initial metastasis** | | |
| Initial metastasis | 14 | 16.9% |
| Initial intracranial metastasis | 2 | 2.4% |
| **WHO tumor grade** | | |
| 2, myxopapillary | 33 | 39.8% |
| 2, non-myxopapillary | 38 | 45.8% |
| 3 | 11 | 13.3% |
| not specified | 1 | 1.2% |
| **DNA methylation group (n=51*)** | | |
| SP-MPE | 32 | 62.7% |
| SP-EPN | 17 | 33.3% |
| SP-EPN-MYCN | 2 | 3.9% |
| **SP-MPE subgroup (n=28**)** | | |
| SP-MPE-A | 18 | 64.3% |
| SP-MPE-B | 9 | 32.1% |
| not attributable | 1 | 3.6% |
| **Extent of resection** | | |
| GTR | 52 | 62.7% |
| <GTR | 22 | 26.5% |
| Biopsy | 9 | 10.8% |
| **Adjuvant treatment** | | |
| Wait-and-see | 46 | 55.4% |
| Radiotherapy | 18 | 21.7% |
| Chemotherapy | 2 | 2.4% |
| Radiochemotherapy | 10 | 12.0% |
| Radioactive seed implantation | 1 | 1.2% |
| Radiochemotherapy followed by secondary GTR | 2 | 2.4% |
| not specified | 4 | 4.8% |
| **Outcome** | | |
| Progression | 26 | 31.3% |
| Death | 4 | 4.8% |

**Supplementary Table S1: Supplementary patient characteristics.** Table includes percentages and further patient characteristics not given in Figure 1. Coccygeal tumor location refers to descriptions of a coccygeal cyst, cystic lesion in the subcutis of the rima ani, or an extradural, extraspinal ependymoma located supracoccygeally. *One patient was excluded due to molecular reclassification of tumor material as anaplastic pilocytic astrocytoma (ANA-PA). **SP-MPE subtyping was impossible in those classified as SP-MPE by immunohistochemistry (n=2) and was missing in n=2 because of unavailable IDAT files. Abbreviations: no. = number, NF2 = *NF2*-related schwannomatosis, SP-MPE = spinal myxopapillary ependymoma, SP-EPN = spinal ependymoma, SP-EPN-MYCN = *MYCN*-amplified SP-EPN, SP-MPE-A = SP-MPE subtype A, SP-MPE-B = SP-MPE subtype B, GTR = gross total resection, <GTR = less than GTR (subtotal or partial resection).


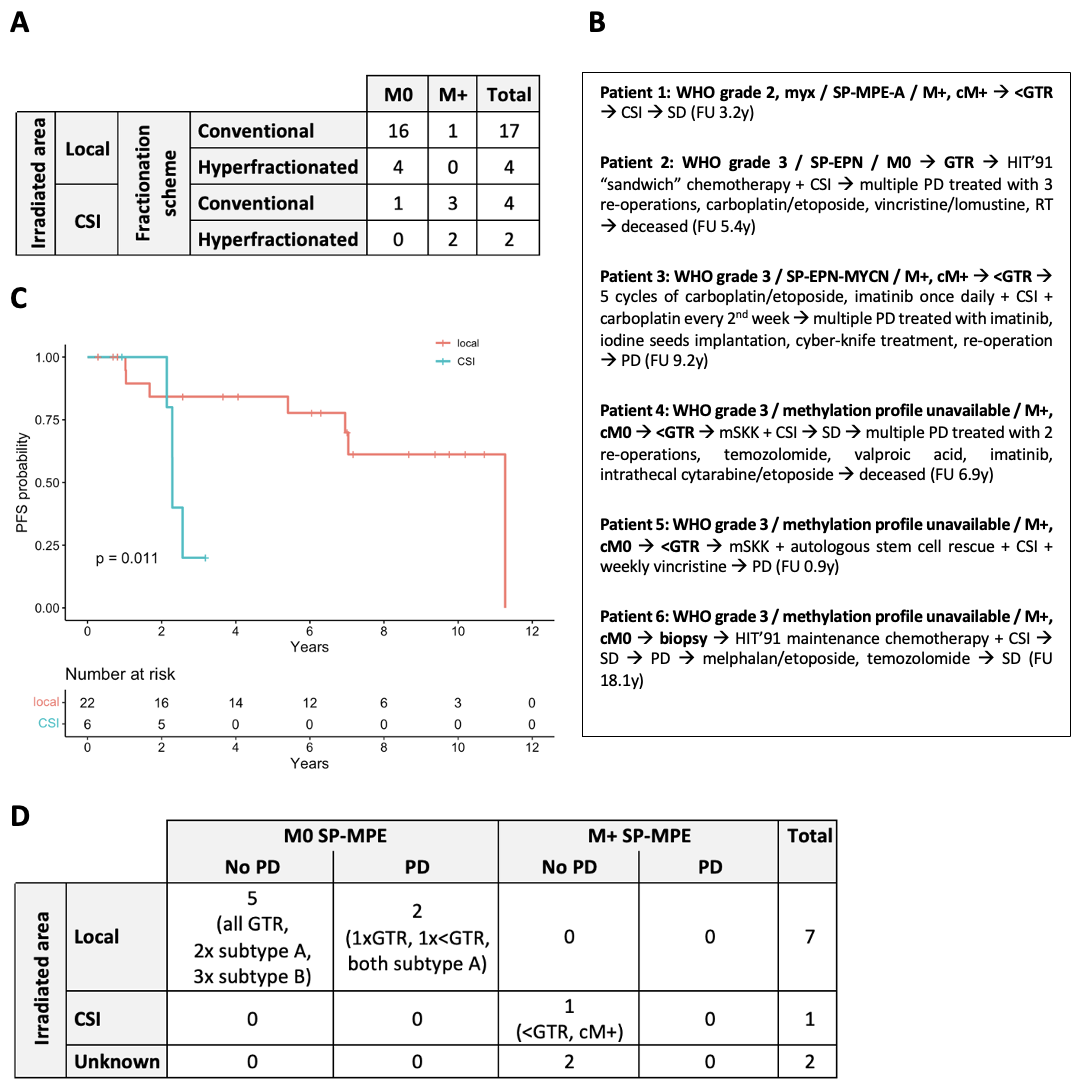


**Supplementary Figure S1: Radiotherapy schemes. (A)** Number of patients with local irradiation or CSI, conventional or hyperfractionated irradiation separated by metastatic status. Irradiated area and fractionation scheme not known in n=2, only fractionation scheme not reported in n=1. **(B)** Clinical course of patients receiving CSI. **(C)** PFS analysis of patients receiving local irradiation vs. CSI, referring to the time to first progression. **(D)** Radiotherapy schemes and outcome in SP-MPE. Abbreviations: CSI = craniospinal irradiation, M0 = no initial dissemination, M+ = initial dissemination, cM+ = initial cranial metastases, cM0 = no initial cranial metastases, myx = myxopapillary, , SP-MPE-A = spinal myxopapillary ependymoma subtype A, SP-EPN = spinal ependymoma, SP-EPN-MYCN = *MYCN*-amplified SP-EPN, GTR = gross total resection, <GTR = less than GTR (subtotal or partial resection), PD = progressive disease/relapse, FU = follow-up time, y = years, mSKK = modified SKK chemotherapy regimen, SD = stable disease, PFS = progression-free survival.

| **Chemotherapy regimens** | **No. of patients** |
| --- | --- |
| Modified HIT-SKK (HIT 2000 Interim protocol) | 3 |
| Modified HIT-SKK with weekly vincristine during radiotherapy (HIT 2000 Interim protocol) | 5 |
| 11 cycles of temozolomide (P-HIT-REZ 2005 protocol) | 1 |
| 12 months of vincristine/carboplatin (HIT-LGG-1996 protocol) | 1 |
| 2 cycles of ifosfamide/etoposide, high-dose methotrexate, cisplatin/cytarabine before radiotherapy (HIT’91 “sandwich” chemotherapy; postradiation chemotherapy not reported) | 1 |
| Weekly vincristine during radiotherapy, followed by 8 cycles of cisplatin/lomustine/vincristine (HIT’91 maintenance chemotherapy) | 1 |
| 5 cycles of carboplatin/etoposide and daily imatinib 400 mg, followed by radiotherapy with concomitant carboplatin every two weeks, followed by maintenance with imatinib for a total of four years | 1 |
| Weekly vincristine during radiotherapy | 1 |

**Supplementary Table S2: Chemotherapy regimens.** One cycle of modified SKK includes one block of cyclophosphamide/vincristine followed by one block of carboplatin/etoposide. Abbreviations: HIT = “Hirntumor “, ie, “brain tumor” in German; SKK = “Säuglinge und Kleinkinder”, ie, “infants and toddlers” in German; LGG = low grade glioma; no. = number.

|  | **5y-OS [%]** | **95%-CI [%]** | **10y-OS [%]** | **95%-CI [%]** | **p** | **5y-PFS [%]** | **95%-CI [%]** | **p** |
| --- | --- | --- | --- | --- | --- | --- | --- | --- |
| **Age** | | | | | | | | |
| <13.5y | 100 | - | 88 | 71.1-100 | n.s. | 60 | 41.5-78.3 | n.s. |
| >13.5y | 100 | - | 83 | 61.7-100 |  | 70 | 54.6-86.2 |  |
| **Sex** | | | | | | | | |
| Male | 100 | - | 94 | 83.6-100 | n.s. | 67 | 50.4-82.8 | n.s. |
| Female | 100 | - | 80 | 58.9-100 |  | 63 | 44.6-81.8 |  |
| **NF2** | | | | | | | | |
| Yes | 100 | - | 100 | - | n.s. | 86 | 59.3-100 | n.s. |
| No | 100 | - | 84 | 68.4-98.8 |  | 62 | 48.4-75.2 |  |
| **Localisation** | | | | | | | | |
| Craniocervical | 100 | - | 100 | - | n.s. | 100 | - | **0.033** |
| Cervical | 100 | - | 100 | - |  | 89 | 67.9-100 |  |
| Cervicothoracic | 100 | - | 83 | 52.9-100 |  | 76 | 46.6-100 |  |
| Thoracic | 100 | - | 67 | 12.3-100 |  | 50 | 0-100 |  |
| Thoracolumbar | 100 | - | 83 | 52.9-100 |  | 42 | 7.1-76.3 |  |
| Lumbar | 100 | - | 100 | - |  | 68 | 45.8-90.2 |  |
| Lumbosacral | 100 | - | 100 | - |  | 44 | 0-88.8 |  |
| Sacral | 100 | - | 100 | - |  | 50 | 0-100 |  |
| Coccygeal | 100 | - | 50 | 0-100 |  | 50 | 0-100 |  |
| **WHO tumor grading** | | | | | | | | |
| 2, myxopapillary | 100 | - | 86 | 59.3-100 | n.s. | 39 | 16.0-62.4 | **0.006** |
| 2, non-myxopapillary | 100 | - | 92 | 77.5-100 |  | 86 | 72.8-99.2 |  |
| 3 | 100 | - | 76 | 46.6-100 |  | 51 | 18.9-82.1 |  |
| **Methylation groups (without SP-EPN-MYCN)** | | | | | | | | |
| SP-MPE | 100 | - | 100 | - | n.s. | 65 | 44.7-85.1 | n.s. |
| SP-EPN | 100 | - | 86 | 59.3-100 |  | 78 | 55.5-100 |  |
| ***MYCN*-amplification** | | | | | | | | |
| *MYCN*-amplified | 100 | - | 50 | 0-100 | n.s. | 0 | - | **0.047** |
| not *MYCN*-amplified | 100 | - | 93 | 80.5-100 |  | 70 | 54.3-85.1 |  |
| **SP-MPE subtypes** | | | | | | | | |
| A | 100 | - | 100 | - | n.s. | 56 | 26.7-85.9 | n.s. |
| B | 100 | - | 100 | - |  | 86 | 59.3-100 |  |

**Supplementary Table S3: Factors influencing OS and PFS.** Abbreviations: OS = overall survival, PFS = progression-free survival, y = year, CI = confidence interval, n.s. = non-significant, NF2 = *NF2*-related schwannomatosis, SP-MPE = spinal myxopapillary ependymoma, SP-EPN = spinal ependymoma.

|  |  | **5y-OS [%]** | **95%-CI [%]** | **10y-OS [%]** | **95%-CI [%]** | **p** | **5y-PFS [%]** | **95%-CI [%]** | **p** |
| --- | --- | --- | --- | --- | --- | --- | --- | --- | --- |
| **Dissemination at diagnosis** | | | | | | | | | |
| Overall cohort | | | | | | | | | |
|  | M0 | 100 | - | 87 | 73.3-100 | n.s. | 67 | 54.2-80.6 | n.s. |
|  | M+ | 100 | - | 80 | 44.2-100 |  | 52 | 20.5-83.7 |  |
| Subgroup: WHO grades | | | | | | | | | |
|  | *Myxopapillary WHO grade 2 tumors* | | | | | | | | |
|  | M0 | 100 | - | 83 | 52.9-100 | n.s. | 31 | 7.0-55.0 | n.s. |
|  | M+ | 100 | - | 100 | - |  | 80 | 44.2-100 |  |
|  | *Non-myxopapillary WHO grade 2 tumors* | | | | | | | | |
|  | M0 | 100 | - | 92 | 75.7-100 | n.s. | 85 | 70.7-99.1 | n.s. |
|  | M+ | 100 | - | 100 | - |  | 100 | - |  |
|  | *WHO grade 3 tumors* | | | | | | | | |
|  | M0 | 83 | 52.9-100 | 83 | 52.9-100 | n.s. | 83 | 52.9-100 | **0.012** |
|  | M+ | 100 | - | 67 | 12.3-100 |  | 0 | - |  |
| Subgroup: methylation groups | | | | | | | | | |
|  | *Subgroup with available methylation profile - overall* | | | | | | | | |
|  | M0 | 100 | - | 77 | 47.0-100 | n.s. | 68 | 50.7-84.7 | n.s. |
|  | M+ | 100 | - | 100 | - |  | 57 | 19.7-94.5 |  |
|  | *SP-MPE* | | | | | | | | |
|  | M0 | 100 | - | 100 | - | n.s. | 64 | 39.6-87.6 | n.s. |
|  | M+ | 100 | - | 100 | - |  | 67 | 28.3-100 |  |
|  | *SP-EPN* | | | | | | | | |
|  | M0 | 100 | - | 86 | 59.3-100 | - | 78 | 55.5-100 | - |
|  | M+ | - | - | - | - |  | - | - |  |
|  | *SP-EPN-MYCN* | | | | | | | | |
|  | M0 | 100 | - | 0 | - | n.s. | 0 | - | n.s. |
|  | M+ | 100 | - | 100 | - |  | 0 | - |  |
| **Extent of resection** | | | | | | | | | |
| Overall cohort | | | | | | | | | |
|  | GTR | 100 | - | 87 | 69.0-100 | n.s. | 75 | 60.3-88.7 | **0.014** |
|  | <GTR | 100 | - | 88 | 64.1-100 |  | 47 | 20.0-74.4 |  |
|  | Biopsy | 100 | - | 80 | 44.2-100 |  | 51 | 14.6-85.4 |  |
| Methylation groups | | | | | | | | | |
|  | *SP-MPE* | | | | | | | | |
|  | GTR | 100 | - | 100 | - | n.s. | 73 | 52.0-93.6 | **0.012** |
|  | <GTR incl. biopsy | 100 | - | 100 | - |  | 25 | 0.0-68.4 |  |
|  | *SP-EPN* | | | | | | | | |
|  | GTR | 100 | - | 67 | 12.3-100 | n.s. | 83 | 52.9-100 | n.s. |
|  | <GTR incl. biopsy | 100 | - | 100 | - |  | 74 | 41.9-100 |  |

**Supplementary Table S4: Impact of initial dissemination and extent of resection on OS and PFS.** Abbreviations: OS = overall survival, PFS = progression-free survival, y = year, CI = confidence interval, n.s. = non-significant, M0 = no initial metastasis, M+ = initial metastasis, SP-MPE = spinal myxopapillary ependymoma, SP-EPN = spinal ependymoma, SP-EPN-MYCN = *MYCN*-amplified SP-EPN, GTR = gross total resection, <GTR = less than GTR (subtotal or partial resection), incl. = inclusively.

|  |  | **5y-OS [%]** | **95%-CI [%]** | **10y-OS [%]** | **95%-CI [%]** | **p** | **5y-PFS [%]** | **95%-CI [%]** | **p** |
| --- | --- | --- | --- | --- | --- | --- | --- | --- | --- |
| **Use of adjuvant treatment (Overall cohort)** | | | | | | | | | |
|  | Adjuvant treatment | 100 | - | 83 | 64.9-100 | n.s. | 75 | 58.6-91.4 | n.s. |
|  | Wait-and-See | 100 | - | 91 | 73.5-100 |  | 55 | 36.5-73.3 |  |
| **Treatment strategies** | | | | | | | | | |
| Overall cohort | | | | | | | | | |
|  | Wait-and-See | 100 | - | 91 | 73.5-100 | n.s. | 55 | 36.5-73.3 | n.s. |
|  | RT | 100 | - | 83 | 52.9-100 |  | 86 | 66.9-100 |  |
|  | CT | 100 | - | 100 | - |  | 100 | - |  |
|  | RCT | 100 | - | 77 | 48.3-100 |  | 55 | 24.5-84.5 |  |
| Treatment strategies in distinct WHO tumor grades | | | | | | | | | |
|  | *Myxopapillary WHO grade 2 tumors* | | | | | | | | |
|  | Wait-and-See | 100 | - | 86 | 59.3-100 | n.s. | 34 | 9.6-58.0 | n.s. |
|  | RT | 100 | - | 100 | - |  | 67 | 12.3-100 |  |
|  | *Non-myxopapillary WHO grade 2 tumors** | | | | | | | | |
|  | Wait-and-See | 100 | - | 100 | - | n.s. | 83 | 60.4-100 | n.s. |
|  | RT | 100 | - | 75 | 31.6-100 |  | 88 | 64.1-100 |  |
|  | CT | 100 | - | 100 | - |  | 100 | - |  |
|  | RCT | 100 | - | 100 | - |  | 80 | 44.2-100 |  |
|  | *WHO grade 3 tumors* | | | | | | | | |
|  | Wait-and-See | 100 | - | 100 | - | n.s. | 0 | - | **0.002**** |
|  | RT | 100 | - | 100 | - |  | 100 | - |  |
|  | RCT | 100 | - | 63 | 19.9-100 |  | 33 | 0-71.1 |  |
| Treatment strategies in distinct methylation groups | | | | | | | | | |
|  | *SP-MPE* | | | | | | | | |
|  | Wait-and-See | 100 | - | 100 | - | n.s. | 51 | 24.5-77.7 | n.s. |
|  | RT | 100 | - | 100 | - |  | 86 | 59.3-100 |  |
|  | RCT | 100 | - | 100 | - |  | 100 | - |  |
|  | *SP-EPN* | | | | | | | | |
|  | Wait-and-See | 100 | - | 100 | - | n.s. | 88 | 64.1-100 | **0.044** |
|  | RT | 100 | - | 100 | - |  | 100 | - |  |
|  | CT | 100 |  | 100 | - |  | 100 | - |  |
|  | RCT | 100 | - | 50 | 0-100 |  | 33 | 0-87.7 |  |
|  | *SP-EPN-MYCN* | | | | | | | | |
|  | RT | 100 | - | 0 | - | n.s. | 0 | - | n.s. |
|  | RCT | 100 | - | 100 | - |  | 0 | - |  |

**Supplementary Table S5: Impact of treatment strategies on OS and PFS.** *not depicted: iodine seeds implantation (n=1). No progressive disease was observed in the patient treated with iodine seeds implantation. **PFS was increased in WHO grade 3 patients treated with adjuvant therapy compared to a wait-and-see approach, yet this finding must be treated with caution as there was only one patient in the wait-and-see group. Abbreviations: OS = overall survival, PFS = progression-free survival, y = year, CI = confidence interval, n.s. = non-significant, RT = radiotherapy, CT = chemotherapy, RCT = radiochemotherapy, SP-MPE = spinal myxopapillary ependymoma, SP-EPN = spinal ependymoma, SP-EPN-MYCN = *MYCN*-amplified SP-EPN.

**
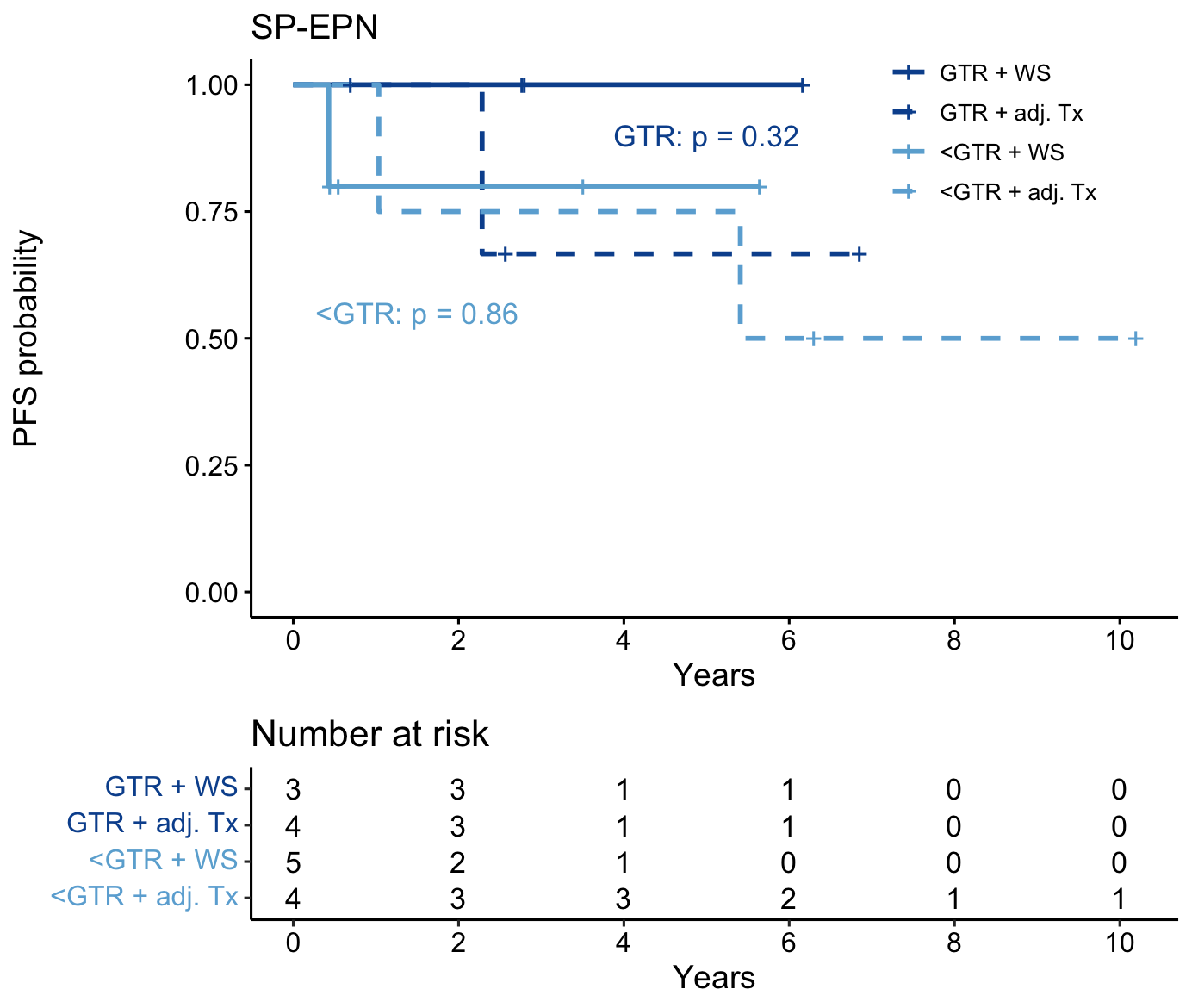
**

**Supplementary Figure S2: PFS analysis for the use of adjuvant treatment in SP-EPN after GTR or <GTR.** Abbreviations: PFS = progression-free survival, SP-EPN = spinal ependymoma, GTR = gross total resection, <GTR = less than GTR (subtotal or partial resection), WS = wait-and-see strategy, adj. Tx = adjuvant treatment.

**Supplementary Figure S3: Treatment of patients with progressive disease.** Abbreviations: PD = progressive disease, GTR = gross total resection, <GTR = less than GTR (subtotal or partial resection), R = residual tumor mass, RT = radiotherapy, CT = chemotherapy, RCT = radiochemotherapy, myx = myxopapillary, SP-MPE = spinal myxopapillary ependymoma, SP-EPN = spinal ependymoma, SP-EPN-MYCN = *MYCN*-amplified SP-EPN.
